# Nanoscale Doping and Its Impact on the Ferroelectric and Piezoelectric Properties of Hf_0.5_Zr_0.5_O_2_

**DOI:** 10.3390/nano12091483

**Published:** 2022-04-27

**Authors:** Anastasia Chouprik, Roman Kirtaev, Evgeny Korostylev, Vitalii Mikheev, Maxim Spiridonov, Dmitrii Negrov

**Affiliations:** Moscow Institute of Physics and Technology, 9 Institutskii Lane, Dolgoprudny 141700, Russia; kirtaev.rv@mipt.ru (R.K.); korostylev.ev@mipt.ru (E.K.); mikheev.vv@phystech.edu (V.M.); spiridonov.mv@mipt.ru (M.S.); negrov.dv@mipt.ru (D.N.)

**Keywords:** ferroelectric hafnium oxide, doping, focused ion beam, piezoelectric coefficient, synchrotron X-ray micro-diffraction

## Abstract

Ferroelectric hafnium oxide thin films—the most promising materials in microelectronics’ non-volatile memory—exhibit both unconventional ferroelectricity and unconventional piezoelectricity. Their exact origin remains controversial, and the relationship between ferroelectric and piezoelectric properties remains unclear. We introduce a new method to investigate this issue, which consists in a local controlled modification of the ferroelectric and piezoelectric properties within a single Hf_0.5_Zr_0.5_O_2_ capacitor device through local doping and a further comparative nanoscopic analysis of the modified regions. By comparing the ferroelectric properties of Ga-doped Hf_0.5_Zr_0.5_O_2_ thin films with the results of piezoresponse force microscopy and their simulation, as well as with the results of in situ synchrotron X-ray microdiffractometry, we demonstrate that, depending on the doping concentration, ferroelectric Hf_0.5_Zr_0.5_O_2_ has either a negative or a positive longitudinal piezoelectric coefficient, and its maximal value is −0.3 pm/V. This is several hundreds or thousands of times less than those of classical ferroelectrics. These changes in piezoelectric properties are accompanied by either improved or decreased remnant polarization, as well as partial or complete domain switching. We conclude that various ferroelectric and piezoelectric properties, and the relationships between them, can be designed for Hf_0.5_Zr_0.5_O_2_ via oxygen vacancies and mechanical-strain engineering, e.g., by doping ferroelectric films.

## 1. Introduction

Doped (or alloyed) HfO_2_-based ferroelectric films have emerged as viable candidates for nonvolatile ferroelectric memories [1] because of their full compatibility with modern silicon microelectronics technology. Many efforts have been focused on the development of three known types of ferroelectric memories based on HfO_2_: ferroelectric random-access memory (FeRAM), ferroelectric field-effect transistors (FeFETs) and ferroelectric tunnel junctions (FTJ). While the performances of HfO_2_-based FeRAM are excellent (except for their retention issues), the benefit of using of this material in FeFET and FTJ is not yet obvious due to poor performances [2]. A common feature of FeFET and FTJ is the origin of the informative signal—the readout signal is determined by the domain structure of the functional ferroelectric layer. Therefore, the memory window in these memories depends not only on the switchable polarization, but also on the fraction of switched domains, as well as the fraction of ferroelectric structural phase in the functional film.

The development of high-performance ferroelectric non-volatile memory devices became mainstream in the field of ferroelectric HfO_2_, while the exploitation of the related piezoelectric properties is another obvious direction. Since high-quality HfO_2_ films can be grown by using the atomic layer deposition technique, even on three-dimensional structures, it is possible to develop piezoelectric devices with new promising designs exploiting either direct or converse piezoelectric effects, e.g., mechanical energy harvesters, self-contained power supplies, transducers, oscillators, etc. In general, these expectations have not yet been met, although some piezoelectric devices were demonstrated [3,4]. The challenge arises from the very small piezoelectric coefficient of hafnium oxide. As reported in a number of publications [5,6,7,8], HfO_2_ doped with different elements has a piezoelectric coefficient of 1–20 pm/V, while in ZrO_2_, it can reach 60 pm/V [9]. For both oxides, it is several tens or hundreds of times less than that of classical ferroelectrics.

Another notable difference between ferroelectric HfO_2_ and classical ferroelectrics is the unusual origin of ferroelectricity, which remains controversial. Recent works predict the crucial role of charged defects in the electric polarization in hafnia. In particular, theoretical calculations have shown that ferroelectricity could originate in oxygen vacancies through electrostrictive effects, which indicates the external nature of polarization switching [10]. In experimental work [11], it was shown that the migration of oxygen vacancies and the associated phase transitions accompany polarization switching in Hf_0.5_Zr_0.5_O_2_ (HZO). The oxygen vacancies are formed through doping by tri- and tetravalent elements [12], as well as through chemical reactions with metal electrodes. High-density non-ferroelectric charges can screen external electric fields and can thus be the external origins of small piezoelectric coefficients. Another known effect of doping is the volume expansion of crystallites due to oxygen vacancies [13], which is one of the causes of the mechanical strain in oxide films [14]. Mechanical-strain engineering is one of the ways to control the ferroelectric (or antiferroelectric) [14] and piezoelectric [15] properties of ferroelectric oxide thin films. However, the effect of doping and the related effects of the non-ferroelectric charges and mechanical strain on the ferroelectricity and piezoelectricity of hafnium oxide, as well as the relationship between ferroelectric and piezoelectric properties, remain unclear [16].

A promising new approach to the investigation of the interplay between ferroelectric and piezoelectric properties is provided by the controlled local modification of the ferroelectric and piezoelectric properties within a single ferroelectric capacitor device and the comparative nanoscopic analysis of the modified regions. Since the ferroelectric and piezoelectric properties strongly depend on doping, local doping with various concentrations by an arbitrary pattern within a single device offers an approach for this type of study. Recently, we reported that the local doping of binary oxide HfO_2_ by Ga ions via their implantation with a low-dose focused ion beam (FIB) into an amorphous film followed by annealing induces local ferroelectricity in HfO_2_ with a high spatial resolution [17,18].

In this work, we demonstrate and employ new and fruitful applications of local doping. Using FIB for the local doping of an initially amorphous Hf_0.5_Zr_0.5_O_2_ (10 nm) film, we reveal that, depending on the doping concentration, the switchable polarization either increases or decreases compared to native HZO. Comparing the ferroelectric properties with the results of in situ piezoresponse force microscopy and the results of a simulation, as well as the results of in situ synchrotron X-ray micro-diffractometry, we demonstrate that changes in switchable polarization are accompanied by changes in the magnitude and sign of the piezoelectric coefficient. The effective piezoelectric coefficient is extremely small and it differs for two monodomain polarizations: −0.30 and −0.15 pm/V for up- and downward polarization, respectively. On the other hand, the doping of HZO films can lead not only to an improvement in the measured switchable polarization, but also to a deterioration in the modulation of the domain structure, i.e., the effects of doping can be opposite for FeRAM and FeFET/FTJ applications.

## 2. Materials and Methods

*Structure fabrication.* Si/TiN/HZO/TiN structures were fabricated in which TiN (40 nm) and TiN (10 nm) grown by magnetron sputtering served as the bottom and top electrodes, respectively. The HZO film 10 nm in thickness was grown via thermal atomic layer deposition at a 240 °C substrate temperature using Hf[N(CH_3_)(C_2_H_5_)]_4_ (TEMAH), Zr[N(CH_3_)(C_2_H_5_)]_4_ (TEMAZ) and H_2_O as precursors and N_2_ as a carrier and a purging gas. Capacitors with dimensions of 50 × 50 μm^2^ were patterned by optical lithography for electrophysical characterization. For the PFM and micro-XRD studies, similar capacitors with dimensions of 100 × 100 μm^2^ were fabricated. Local crystallization of the HZO films into ferroelectric phase occurred after Ga implantation during post-metallization rapid thermal annealing for 30 s at 500 °C in Ar. For studies of the impact of post crystallization Ga implantation, some capacitors were Ga-irradiated after thermal annealing. For the PFM and micro-XRD studies, the functional capacitors were routed to the Al contact pads, allowing external electric biasing of the capacitors. The routing fabrication details were described previously [19].

*Gallium implantation* was performed using the dual-beam system Jeol JIB4501 (scanning electron microscope with LaB6 emitter, FIB with Ga liquid metal ion source). The 30 kV, 100 pA electron beam, the 30 kV, 50 pA ion beam, and the sample surface were aligned to eucentric height. Next, only the e-beam was used for navigation and positioning to the region of interest; no ion beam image scans were performed. After selection of a suitable region by e-beam imaging, ion-beam patterning was performed at a 0° incident angle. Ion beam was focused to a spot ~40 nm in diameter. Prior to implanting gallium ions, this process was simulated using binary collision approximation (SRIM/TRIM software) by means of three-layer model of TiN/HZO/TiN stack (details of simulation are described in Supporting Information, Section S1). Eight capacitors were fabricated for every Ga concentration of 0.1, 0.3, 0.5, 0.7, 0.9, 1.1, 1.3, 1.5, and 2.0 at.%.

For *electrophysical characterization*, the Cascade probe station coupled with semiconductor parameter analyzer Agilent B1500A was used. *P–V* curves were measured through the dynamic positive-up negative-down (PUND)–like technique [19]. To wake up the as-prepared HZO film, the ferroelectric capacitors were cycled 10^5^ times by applying bipolar voltage double triangular pulses with an amplitude of ±3.5 V and a duration of 100 μs. Dielectric permittivity was acquired for cycled structures through *C–V* curves measured at an excitation voltage of 10 kHz, 50 mV and series capacitor–series resistor (*C*s-*R*s)-equivalent scheme. 

*Piezoresponse force microscopy.* Local piezoresponse was characterized via the in-house-implemented resonance-enhanced band excitation PFM technique [20] using an Ntegra atomic force microscope (NT-MDT, Russia) and a Keysight M3300A arbitrary waveform generator/digitizer. The experimental scheme and the details of the BE PFM were described previously [19,21]. To minimize the contribution of the parasitic electrostatic tip–surface interactions, the PFM experiments were carried out at the patterned capacitors routed to the contact pads. All measurements were performed from the woken-up capacitors to ensure the stability of the ferroelectric and piezoelectric properties. 

The procedure for calibration of the PFM amplitude to piezoelectric coefficient using thermal noise spectra is described in Supporting Information, Section S4.

The electrical excitation of the ferroelectric layer was performed with the following waveform parameters: the central frequency near the contact resonance frequency was ~560 kHz, the bandwidth was 100 kHz with 1024 frequency bins, and the peak-to-peak value of exciting voltage was 0.8 V [21]. Composite poly-Si&Si cantilevers HA_FM Etalon (ScanSens, Bremen, Germany) with a free resonance frequency of 110 kHz and a force constant of 6 N/m were used in our experiment. The loading force was constant and equaled ~150 nN. 

*Simulation of the PFM results*. The displacement of the surface of the top capacitor electrode interpreted as the PFM results was simulated by the finite-element analysis using equations of solid mechanics physics at the Comsol software. We used the Comsol database to define materials. The geometry, boundary conditions, and distribution of the static uniform load (simulated domains) are shown in Figure 4.

*In situ synchrotron X-ray microdiffractometry.* The measurement of the piezoelectric coefficient by means of in situ XRD using synchrotron radiation was performed at beamline P23 in the DESY synchrotron research facility, Germany. Notably, the X-ray beam was focused on the sample surface to a spot approximately 30 μm in diameter, which was less than the size of the top electrodes in our capacitor structure. Bottom electrode was grounded during the experiment, while the bias was applied to the top electrode. All measurements were performed from the woken-up capacitors to eliminate the possible contributions of the crystal structure changes by the application of bias.

## 3. Results and Discussion

The effect of doping on the ferroelectric and piezoelectric properties of the HZO (10 nm) was determined in two steps. In the first step, we elucidated the effect of doping with different Ga concentrations (at.%) on the switchable polarization and internal bias fields. In the second step, the local ferroelectric and piezoelectric properties of the regions doped with selected Ga concentrations were revealed, including the modulation of the domain structure by the switching voltage, as well as the piezoelectric coefficient value. 

### 3.1. Ferroelectric Properties of Ga-Doped HZO Capacitors

**Figure 1:**
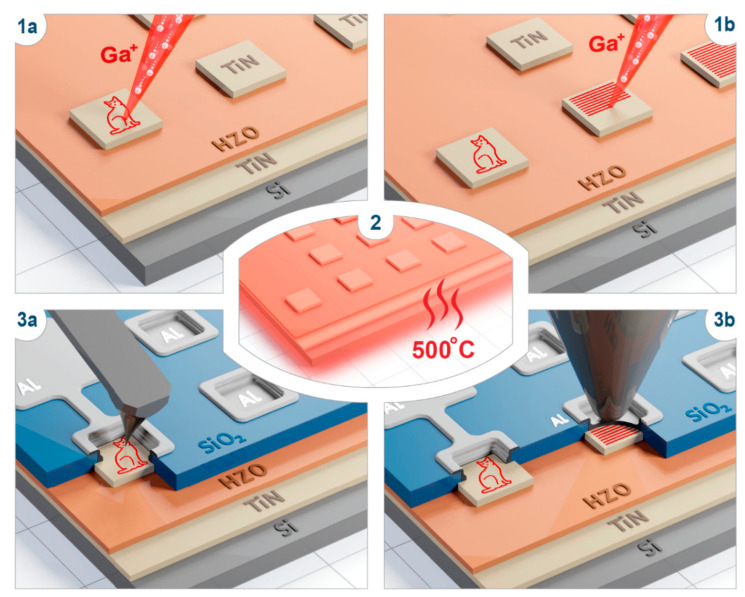
Design of the experiment. First step: The focused ion beam raster scans across the capacitor, implanting Ga ions either (**1a**) locally or (**1b**) homogeneously. Second (**2**) step: annealing takes place for crystallization of HZO (HZO) either into the ferroelectric phase or into the polymorphic state. Third step: Capacitors are addressed microscopically and macroscopically, either by a PFM study (**3a**) or electrophysical characterization at a probe station (**3b**).

For the study of the ferroelectric properties, the capacitors Si/TiN (40 nm)/HZO (10 nm)/TiN(10 nm) 50 × 50 µm^2^ in size with *homogeneous* Ga doping were fabricated. Homogeneous doping was performed by raster FIB scanning over the *whole* capacitor area, over the top electrode, as it is schematically shown in Figure 1 (details of the structure fabrication and Ga implantation are described in the Section 2). The ion doses required to obtain various concentrations of Ga in HfO_2_ were calculated using a Monte Carlo simulation of the motion of the ions in condensed matter (details are described in the Supporting Information, Section S1) and ranged from zero (native film) to 1.2 × 10^15^ ion/cm^2^ (which corresponds to 1.5 at.%). For sufficient statistics, eight capacitors were fabricated for each Ga concentration. The crystallization of the Ga-doped HZO films was induced by subsequent rapid thermal annealing at 500 °C.

**Figure 2:**
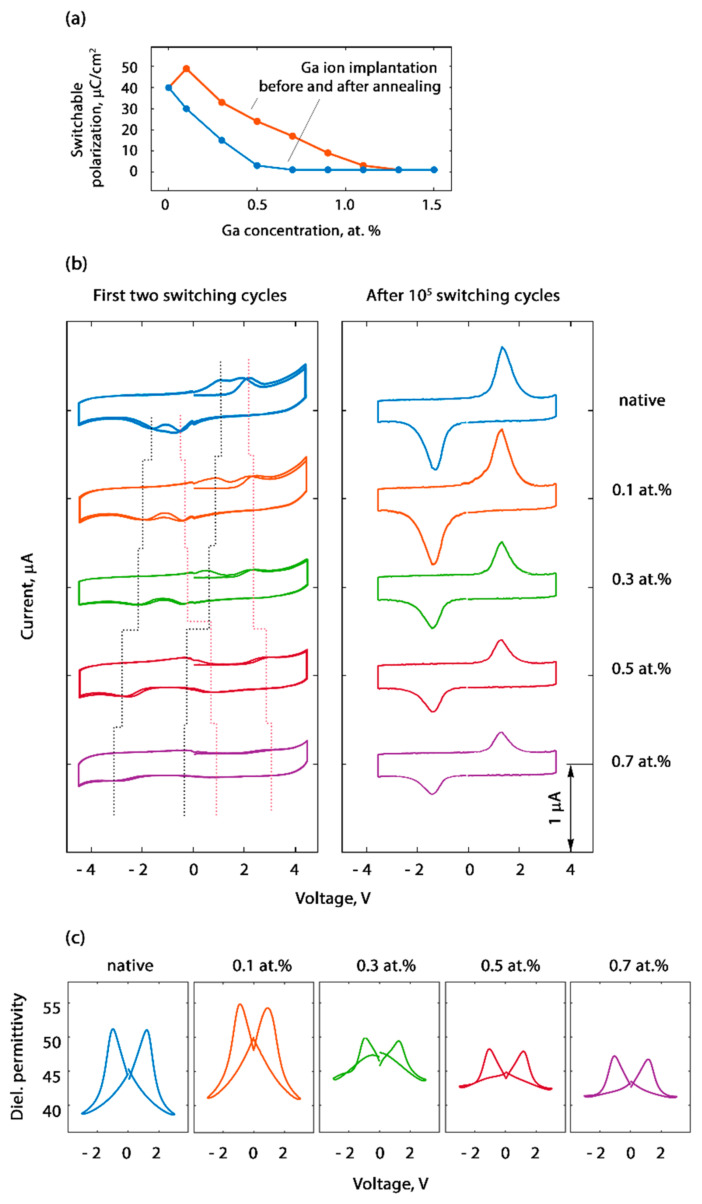
Electrophysical properties of homogeneously doped HZO capacitors: (**a**) Dependences of the switchable polarization on the Ga concentration for ion implantation performed before or after crystallization; switchable polarization obtained after 10^5^ switching cycles. (**b**) Switching *I–V* curves measured during the first two switching cycles and after 10^5^ switching cycles for selected Ga concentrations. Dotted lines indicate the positions of the current peaks. (**c**) *C–V* curves measured after 10^5^ switching cycles.

The maximal switchable polarization of 51 μC/cm^2^ was achieved at a Ga concentration of 0.1 at.% (Figure 2a), while the native HZO film exhibited a switchable polarization of 40 μC/cm^2^. A further increase in the Ga concentration resulted in a decrease in the measured switchable polarization. These results are in line with the results for the HZO doped with La during the atomic layer deposition and annealed at the same temperature [22]. The switchable polarization values were obtained after 10^5^ bipolar switching cycles, performed to stabilize the ferroelectric properties of the capacitors. The so-called wake-up effect, consisting in the stabilization of the ferroelectric properties after the first thousand switching cycles, is a peculiar property of ferroelectric HfO_2_-based capacitors. Usually, the wake-up effect was associated with an increase in the fraction of the switching domains due to the electrically driven redistribution of the charged oxygen vacancies that initially accumulated at the electrode interfaces and pinned the domains [23].

Another similarity between the Ga- and La-doped HZO was the manifestation of antiferroelectric-like behavior at high Ga concentrations. All the fresh capacitors demonstrated splitting of the switching *I–V* curves. The distance between the positions of the current peaks was larger at higher Ga concentrations (Figure 2b). Usually, the shape of the switching *I–V* curves was associated with the distribution of the internal bias fields. Thus, the antiferroelectric-like splitting of the switching *I–V* curves was attributed to the existence of two populations of domains that appeared during the crystallization process and subsequently produced two opposite internal bias fields due to the opposite orientation of the vertical component of the polarization vector [24]. From this standpoint, higher Ga concentrations caused larger internal bias fields and, therefore, a larger number of switching cycles was required to transfer the structure into the ferroelectric state.

Another possible reason for the splitting of the *I–V* curves may have been the manifestation of the antiferroelectric tetragonal structural phase of the HZO, which correlated with the dielectric permittivity. The dielectric permittivity *k* increased with the Ga concentration in the range 0.1–0.3 at.% (Figure 2c), which indicates that the stabilization of the tetragonal phase was quite probable. Indeed, for the orthorhombic HfO_2_ phase *Pca*2_1_, *k* varied within the range of 27–35 depending on the spatial orientation of the grains, whereas for the antiferroelectric tetragonal *P*4_2_/*nmc* phase *k* = 28–70 [25]. A further decrease in the dielectric permittivity with an increase in the Ga concentration was associated with the appearance of a paraelectric monoclinic *P*2_1_/c phase or an amorphous state (*k* = 15–20).

It is noteworthy that the small-signal *C–V* curves of the cycled HZO:Ga capacitors contained some antiferroelectric-like contribution even after the wake-up, when the *I–V* curves seemed to be purely ferroelectric. This means that the ferroelectric properties of the doped HZO were quite unusual.

While the Ga doping performed before the crystallization of the film determined the structural phase composition of the HZO that appeared during annealing, the implantation of the Ga ions into the already crystallized film caused another effect. We explored the effect of introducing defects into ferroelectric HZO on its ferroelectric properties, both locally and area-averaged. The testing of the homogeneously modified HZO revealed a deterioration in the measured switchable polarization of the capacitors with the post-crystallization implantation of the Ga ions (Figure 2a), which was in line with the results for the PbZr_0.1_Ti_0.9_O_3_ (PZT) [26].

### 3.2. Local Ferroelectric and Piezoelectric Properties of Native and Doped HZO

**Figure 3:**
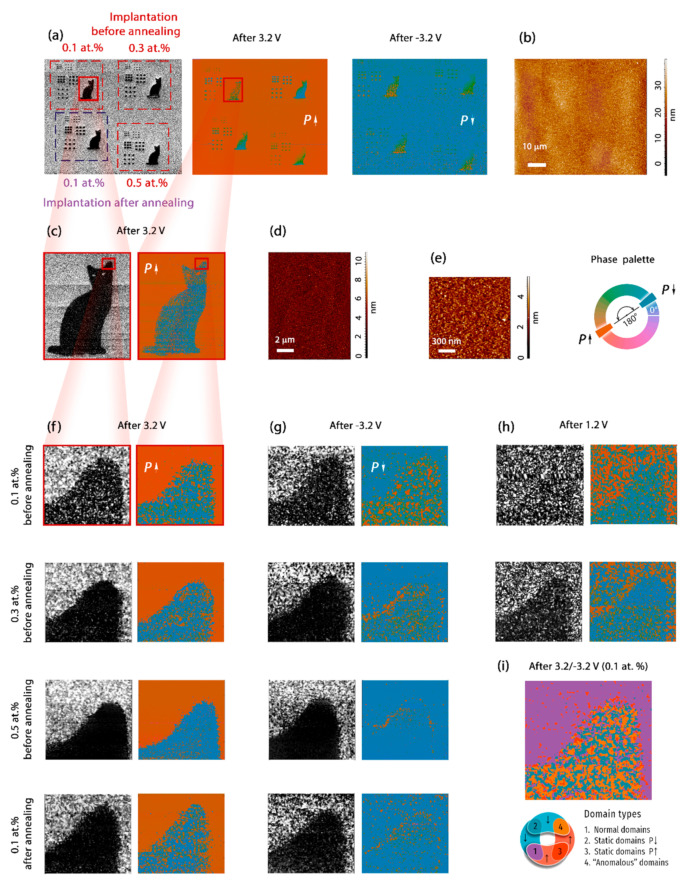
Domain switching within native HZO and regions doped with various Ga concentrations; gray-colored maps are the PFM amplitude maps, orange-blue maps are the PFM phase maps. The phase palette is common to all the PFM phase maps. (**a**) Domain structure at low magnification. (**b**) Topography of the top TiN electrode at low magnification. (**c**) Domain structure at medium magnification. (**d**,**e**) Topography of the top TiN electrode at medium and high magnification. (**f**–**h**) Domain structure at high magnification of the regions doped with some Ga concentrations after applying 3.2, −3.2 and 1.2 V. (**i**) Identification of four types of domain within the region doped with 0.1 at.% (obtained by overlaying the PFM-phase maps in (**f**,**g**)).

After revealing the effect of the doping on the ferroelectric properties of the homogeneously doped films, the ferroelectric properties of the locally doped HZO were investigated. For this purpose, the capacitors with the top electrodes routed to the external pads for electric biasing were fabricated (as shown in Figure 1; details the fabrication of the routed capacitors were described previously [19]). For the most reliable analysis of the effect of the concentration on the local ferroelectric and piezoelectric properties, four regions with different concentrations and with either the pre- or post-annealing implantation of Ga ions were fabricated within the same capacitor. Three regions contained patterns of Ga doping performed before the annealing (with 0.1, 0.3, 0.5 at.%), whereas in the fourth region, the implantation was performed after the HZO crystallization (with 0.1 at.%) (Figure 3a).

Piezoresponse force microscopy (PFM) was employed (details are described in the Section 2) to visualize the magnitude of the local piezoelectric response and the orientation of the polarization vector in the capacitor, which was preliminarily subjected to 10^5^ switching cycles. Only miniscule changes (~0.3 nm in height) were detectable in the Ga-irradiated regions during careful inspection of the morphology maps (Figure 3b–e), i.e., the modification of the top TiN layer by the focused ion beam was very minor. We will now analyze the PFM results.

It is known that, fundamentally, the PFM technique provides two informative quantities: The PFM amplitude, associated with the absolute magnitude of the effective longitudinal piezoelectric coefficient *d*_33_*; and the PFM phase, associated either with the orientation of the polarization vector or with the sign of the piezoelectric coefficient *d*_33_*. Even a cursory analysis of the overview maps of the PFM amplitude (Figure 3a) reveals a smaller value of the effective piezoelectric coefficient in the doped region (compared to the native HZO), including in the region with the Ga concentration of 0.1 at.%, which showed an improvement in the switchable polarization. Meanwhile, in classical ferroelectrics, the piezoelectric coefficient is proportionally related to the remnant polarization *P*_s_ (*d*_33_ = 2ε_0_*kQ*_11_*P*_s_, where ε_0_ is the permittivity of the vacuum and *Q*_33_ is the electrostrictive constant), and a larger piezoresponse should be expected for lightly doped HZO given equal *Q*_11_. A cursory review of the PFM-phase maps shows that the native (undoped) HZO film demonstrated almost full switching of the PFM phase, i.e., full polarization reversal, as a result of applying a voltage pulse of either 3.2 or −3.2 V (1 ms) to the capacitor plates (Figure 3a). This observation is consistent with previous results for HZO-based capacitors [19]. The switching of the domain structure within the doped regions was not as obvious. Therefore, both the behavior of the piezoelectric coefficient and the orientation of the polarization vector should be analyzed in more detail. We start with the analysis of the regions doped *before* the crystallization process, and then compare them with the region irradiated *after* the annealing.

#### 3.2.1. Qualitative Analysis of the Magnitude of the Piezoresponse Determined by the Local Piezoelectric Coefficient

The first microscopic finding was a decrease in the magnitude of the piezoresponse with the dopant concentration (Figure 3f,g). Since this quantity is associated with the effective piezoelectric coefficient *d*_33_*, it can be concluded that *d*_33_* decreased with the dopant concentration. This result was expected for dopant concentrations of 0.3 and 0.5 at.%, which showed decreased switchable polarization. The surprising result came from the region doped with 0.1 at.%., which demonstrated improved remnant polarization and, at the same time, decreased piezoelectric coefficient compared with the native HZO. 

A more careful inspection of the PFM phase maps revealed a polydomain structure within the doped region 0.1 at.% (upper line in Figure 3f,g,i). Notably, in the polydomain state, adjacent domains with opposite orientations of the vertical component of the polarization vector wee mechanically coupled to each other since the top electrode layer covered them. As a result, the displacement of the surface of the top electrode caused by the converse piezoelectric effect and recalculated into the local piezoelectric coefficient was decreased compared with that of the monodomain state.

**Figure 4:**
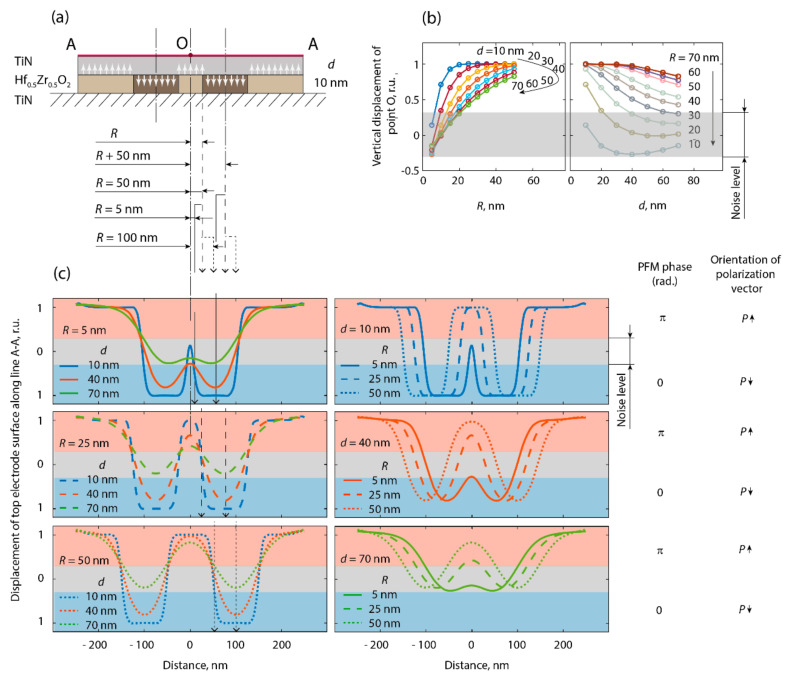
On the spatial resolution of PFM of capacitor devices: (**a**) geometry for the finite element simulation, **(b**) calculated dependences of relative vertical displacement of central point of top electrode above a single domain (point O) on domain size and thickness of top electrode, (**c**) calculated relative displacement of the surface of top electrode (line A–A) at different lateral sizes of the central domain and thickness of top electrode. Displacements of 0–1 and 0–1 r.u. correspond to the PFM phase of π and 0 and thus to the up- and downward orientation of vertical component of polarization, respectively. Orange and blue coloring correspond to similar colors on PFM-phase maps. Gray color indicates a noise range.

To obtain an insight into the impact of the electrode layer on the measured piezoelectric coefficient of thin-film devices, we simulated the vertical displacement of the surface of the top electrode above a ferroelectric film in the polydomain state. The geometry and force distribution emulating the piezoelectric deformation of the HZO in the finite element analysis are shown in Figure 4. To determine the role of the top electrode in the piezoelectric coefficient measured in PFM, we varied two parameters: the thickness of the top electrode and the lateral size of a single domain (the central domain in Figure 4a). 

It was found that the vertical displacement of the surface above the single domain reached a maximal value that corresponded to the genuine piezoelectric coefficient only if the single domain was wider than ~50 nm, while for the smaller domains, the measured PFM amplitude was suppressed. Even if the top electrode is very thin (e.g., 10 nm in thickness, like in our experiment), a small domain (e.g., ~10 nm in diameter) has a similar appearance to a non-piezoelectric region. Wider domains (20–40 nm in diameter) were distinguishable in the PFM, including their orientation of the polarization vector; however, the magnitude of the piezoresponse associated with the local piezoelectric coefficient was suppressed. The described effect was not related to the PFM apparatus and it was not a PFM artifact—it was a pure mechanical effect due to the mechanical coupling between adjacent domains by the passive top electrode layer. A sharp AFM tip served as a sensor of the surface displacement and it precisely detected this displacement.

If we consider a polycrystalline HZO film, it should be noted that each grain is a separate domain, with a certain orientation of its polar axis. The width of grains varies in the range 5–50 nm, and grains typically span the whole thickness of the film [19]. In polydomain capacitors with top electrodes thicker than 20 nm, the surface displacement is always suppressed and the measured piezoelectric coefficient is decreased. 

Since the region with 0.1 at.% consisted of a polydomain structure, even after applying either −3.2 or 3.2 V, its smaller piezoelectric coefficient was probably associated with the constricted displacement of the top electrode. This assumption was confirmed by comparing the magnitude of the piezoresponse in the native HZO region and the Ga-doped (0.1 at.%) region when they were both in the polydomain state, which was set after applying of a mean coercive voltage (1.2 V). It turned out that their domain structures were qualitatively and quantitatively similar. (Figure 3b) The shapes and sizes of the domains, as well as the magnitude of the piezoresponse, were approximately the same, although the mutual fractions of the domains were opposite each other (Figure 3b). On the one hand, the typical domain structure was a signature of the genuine ferroelectric properties of the Ga-doped HZO. On the other hand, this observation confirms that the magnitude of the piezoelectric coefficient measured within the regions of the doped HZO was limited by the effect of mechanical coupling of the adjacent domains by the passive layer of the top electrode. 

At higher Ga concentrations, the domains in the polydomain state are seemed to become less prominent (Figure 3h). At 0.3 at.%, the magnitude of the piezoresponse decreased almost to the PFM noise level, and at 0.5 at.%, the magnitude became so small that the PFM phase was fully determined by the non-ideally compensated background of our PFM setup (not shown). Since, on the one hand, (i) the switching *I–V* curves clearly exhibit the switching of the polarization for the homogeneously doped HZO with Ga concentrations of 0.1, 0.3 and 0.5 at.%, (ii) the switchable polarization gradually decreased with the concentration, and, on the other hand, (iii) the Ga-doped HZO with 0.1 at.% clearly exhibited the polydomain structure after 3.2 and −3.2 V, we assume that the Ga-doped HZO regions with 0.3 and 0.5 at.% of Ga containing the mixture of ferroelectric and non-ferroelectric structural phases and domains in ferroelectric regions possibly decreased compared with native the HZO and HZO doped with 0.1 at.% of Ga. Taking into account the effect of mechanical coupling in thin-film capacitor devices, only this explanation can satisfy all the experimental results. 

In the region of the post-annealing implantation of the Ga ions, as expected, a simple pinning of domains was observed during the capacitor switching (Figure 3g,h). This result was consistent with the decrease in the measured switchable polarization (Figure 2a), and it was in line with a similar experiment on defect engineering in a ferroelectric PZT film [26].

In addition to the described dependence of the magnitude of the piezoresponse on the dopant concentration, we revealed a specific effect related to the sign of the piezoresponse.

#### 3.2.2. Sign of the Piezoresponse Determined either by the Orientation of the Vertical Component of the Polarization Vector or the Sign of the Piezoelectric Coefficient

**Figure 5:**
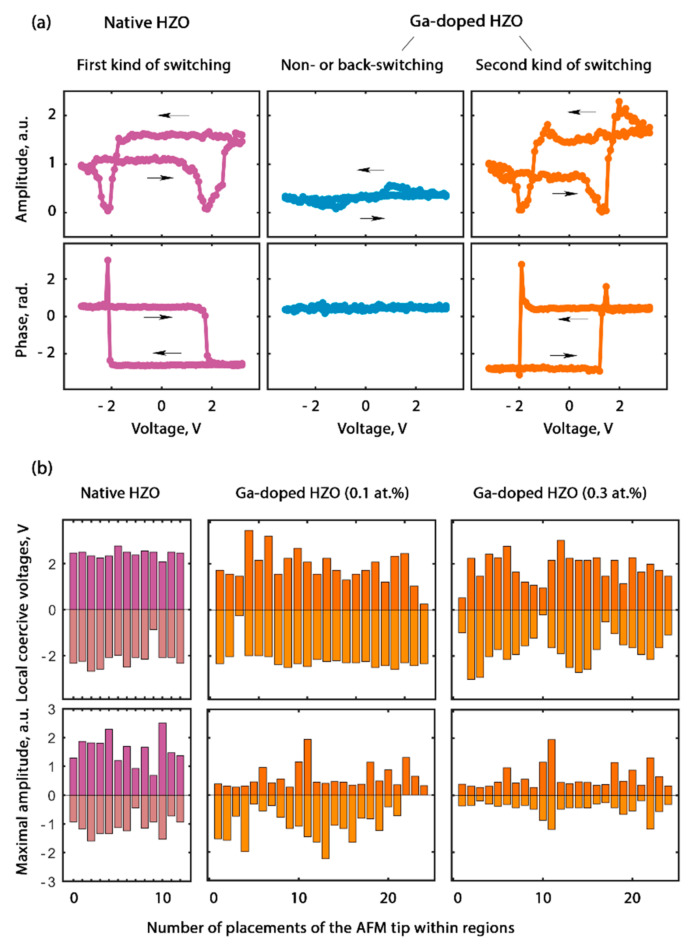
Local SS–PFM loops measured within the native and doped HZO regions: (**a**) three types of loop (the left curves are typical for native HZO, while the middle and right curves are both typical for HZO doped with 0.1 at.%). (**b**) Local coercive voltages and maximal amplitude during the local switching in a number of AFM tip locations within different HZO regions.

A more careful analysis of the PFM-phase maps revealed an opposite switching of the PFM phase in the native and doped regions. The overlaying of two PFM-phase maps made clear that the PFM phase in the native HZO film switched uniformly, while in the doped film, four types of domain were observed (Figure 3i). Most of the doped area consisted of non-switching domains and “anomalous” domains, in which the PFM phase switched opposite to the PFM phase in the native HZO. We studied this effect in more detail. The off-field single-switching band excitation piezoresponse force microscopy (SS–BE–PFM; SS–PFM; the voltage train is presented in Supporting Information, Section S2) was used to study the local switching of both the magnitude and the sign of the piezoresponse in both the native and the doped regions. At any location of the AFM tip above the native film, the phase switched by 180°, which was attributed to the polarization reversal (left panel in Figure 5a). In the region doped with 0.1 at.%, two further types of behavior of the PFM phase were observed. On some of the SS–PFM curves, the phase did not change (middle panel in Figure 5a), i.e., the vertical component of the polarization did not switch. Other curves demonstrated the PFM phase switching by 180°; however, this switching was 180° out of phase relative to the curves measured on the native film (cf. right and left panels in Figure 5a). A statistical analysis of a number of SS–PFM curves in different regions confirmed the decrease in the piezoresponse in all the doped regions. The coercive voltages did not vary with the doping, which was in line with the *P–V* curves (Figure 2b). 

The results of the study of the local switching confirmed an opposite PFM-phase switching in the native and doped HZO, as revealed during the PFM mapping. In the piezoelectric coefficient has the same sign in all regions, this may mean that the PFM phase switches anomalously in either native or doped region.

Let us discuss the possible causes of the phenomenon of the anomalous switching of the PFM phase. First, the PFM artifacts (e.g., associated with electrostatic contribution) could have caused the PFM phase to change. Second, two kinds of PFM phase switching could be associated with normal polarization switching in one region and genuine anomalous polarization switching in other region. This means that after passing the voltage pulse with some threshold amplitude, the polarization vector of the grain under the AFM tip aligned either *along* or *against* the applied field and stayed this way after the field was turned off. Third, ferroelastic switching, accompanied by non-180° rotation of the polarization vector [27,28], could have induced an unexpected orientation of the vertical component of the polarization vector after the capacitor switching. Fourth, the opposite switching of the piezoresponse in adjacent regions could be associated with the opposite sign of their piezoelectric coefficient. For example, the native HZO may have had a positive piezoelectric coefficient, while the doped HZO may have had a negative piezoelectric coefficient, or vice versa. 

**Figure 6:**
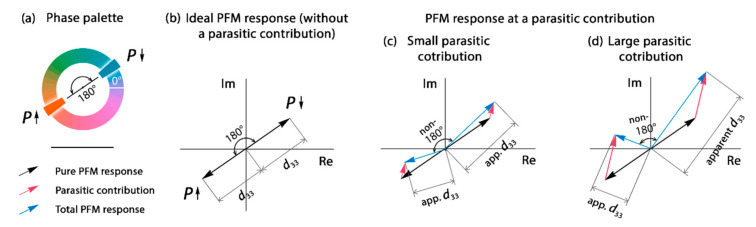
Complex PFM response for two polarization states and variations in the measured PFM phase and PFM amplitude at different conditions. (**a**) Phase palette used in Figure 3; (**b**) Ideal PFM response in the absence of parasitic contribution; PFM response at (**c**) small and (**d**) large parasitic contribution. Notation “*d*_33_” means real *d*_33_ that was measured in PFM given the absence of a parasitic contribution. Notation “apparent *d*_33_” (“app. *d*_33_”) corresponds to the *d*_33_ measured when a parasitic contribution was present.

The use of PFM artifacts is a broad topic, so our aim is to distill the information relevant to this particular experiment. The experiment was designed in such a way as to minimize artifacts. In contrast to the PFM study of the bare ferroelectric film, the study of the ferroelectric capacitor device canceled out the issues related to the parasitic injection of charge due to the enhancement of the electric field by a sharp AFM tip, as well as to the electrochemical reactions on the bare ferroelectric surface and the screening of the polarization charge by charges from the ambient environment. Any possible charge injection/trapping and electrochemical issues totally corresponded to these phenomena in the real capacitor device during its operation, and, thus, the PFM results corresponded to the phenomena that occurred inside the device. However, an injection occurring in electronic devices can potentially change the PFM phase during the study of the device. In a good PFM setup (with a subtracted background), the domains with the opposite vertical component of the polarization vector exhibit 180° phase difference in the complex piezoresponse (Figure 6a), given the absence of parasitic charging. If any parasitic charge (including the charge trapped by the defects at the electrode interfaces) contributes to the measured response, then the complex piezoresponse and the complex parasitic component are summed, and the difference in the PFM phase decreases. The deviation of the phase difference is as large as the parasitic contribution (in Figure 6b, small and large constant parasitic contributions are shown). Therefore, the deviation of the phase difference from 180° is always a signature of a PFM artifact, and vice versa—an exact phase difference of 180° (as in our experiment) is a signature of reliable experimental results. It should also be noted that any charging/discharging effects are time-dependent. If they had contributed to the PFM results, then the piezoresponse maps would have evolved with time [29]. As a result, the vectors of the total response would have begun to rotate, and the phase difference would have begun to develop. However, no temporal evolution was observed in the phase difference.

Anomalous polarization switching was the most likely explanation for the anomalous switching of the PFM phase observed upon the wake-up of the native HZO [23]. This explanation was based on (i) the polarization back-switching observed on the *P–V* curves during the wake-up, (ii) a very high concentration of traps at the electrode interface (up to 10^21^ cm^−3^), and (iii) no temporal evolution of the PFM phase difference. Under such conditions, the high-density interface charge trapped during the switching pulse could cause the back-switching of a part of the polarization during the falling slope of the voltage pulse at *V* → 0 or after the voltage pulse passed. However, the homogeneously Ga-doped HZO capacitors subjected to the wake-up procedure demonstrated only a minor polarization back-switching (Figure 2b) and no depolarization was observed on a series of sequentially measured macroscopical *P–V* curves. 

Ferroelastic switching due to the coupling between the applied electric field and the mechanical strain can also cause the vertical polarization component to align against the applied field. The mechanical strain can originate from the volume expansion of crystallites due to the oxygen vacancies [13,14] formed due to doping [12]. Lederer et al. reported that ferroelastic switching could also be responsible for the antiferroelectric-like behavior and splitting of switching *I–V* curves during wake-up [28]. This is reminiscent of the antiferroelectric features of the properties of the doped HZO, namely, the aggravation of both the splitting of the switching *I–V* curves with the Ga concentration and the antiferroelectric-like contribution to the *C–V* curves (Figure 2b,c). Another argument for the ferroelastic switching in the doped region is the self-crossing of the PFM amplitude loops, visible in the range of coercive voltage (Figure 5a), because it is specific to ferroelastic switching [30,31]. A non-180° switching of the polarization vector due to the ferroelastic effect could mimic the piezoelectric effect with the opposite sign of the piezoelectric coefficient. From the standpoint of piezoelectric applications, these two causes of opposite PFM-phase switching are formally similar to each other. The piezoelectric coefficient of the different signs in the native and doped regions is one of the main possible reasons for the observed switching of the piezoresponse.

In general, ten percent of piezoelectrics have a negative piezoelectric coefficient [32], i.e., the negative piezoelectric coefficient of hafnia is not out of the ordinary. It has recently been reported that the ferroelectric hafnia is able to exhibit a negative piezoelectric coefficient, and doping plays a crucial role [6,7,15]. Dutta et al. [15] reported that HfO_2_:La showed 180° out-of-phase switching of the PFM phase compared to HZO and HfO_2_:Gd. After the calibration of the PFM phase, they concluded that HZO and HfO_2_:Gd have a positive piezoelectric coefficient, whereas HfO_2_:La has a negative piezoelectric coefficient. The authors also predicted that the ferroelectric phase of the hafnia would be modified via a strain and, thus, different magnitudes and signs of the piezoelectric coefficient would be achieved. Once both positive and negative piezoelectric coefficients can be achieved in the same material, under certain conditions, they are equal to zero, which is reminiscent of the decreased magnitude of piezoresponse within the highly doped HZO (Figure 3).

It is noteworthy that the sign of the piezoelectric coefficient measured by the PFM depends on the calibration of the PFM phase. Usually, it is conducted using calibration samples with reliably known properties, e.g., a commercially available crystal of periodically poled lithium niobate. However, such a calibration is itself a potential source of artifacts, because the PFM phase depends on the sample and the scheme of the PFM experiment. Since hafnia can have any sign of the piezoelectric coefficient, we performed the most straightforward calibration of the piezoelectric coefficient; specifically, we measured the piezoelectric coefficient of the HZO by means of in situ synchrotron X-ray microdiffractometry.

### 3.3. Quantitative Measurement of the Magnitude and Sign of the Piezoelectric Coefficient

#### 3.3.1. The Piezoelectric Coefficient Measured by Means of In Situ Synchrotron X-ray Microdiffractometry

For the measurement of the piezoelectric coefficient of native HZO by in situ synchrotron X-ray microdiffractometry (micro-XRD), we used the sample with routed capacitors 100 μm in size, i.e., the XRD sample was very similar to the PFM sample. After the wake-up procedure, we applied the negative voltage (−3 V, 1 ms) to the top electrode to induce polarization reversal in the upward direction in the ferroelectric capacitor based on the native HZO film. Next, we applied sequential bias voltage, −0.5, 0, 0.5 V and, once more, 0 V, to the top electrode. Note that 0.5 V is lower than the coercive voltage of the HZO. Thus, we did not expect polarization reversal in the downward direction during the experiment. The in situ measurements of the XRD spectrum performed simultaneously with external bias variation were expected to reflect the variation in the lattice parameters (including *d*-spacing) in the upward polarization state with bias change. A similar measurement was carried out after the polarization reversal in the downward direction by the application of 3 V. Notably, the X-ray beam spot was approximately 30 μm in diameter, which was less than the size of the top electrodes in our capacitor structure. Thus, only the XRD from the HZO located under the top electrode was ensured. 

The simplest way to estimate the effective piezoelectric coefficient along the normal surface direction was to analyze the evolution of the *d*-spacing between the (002) planes of the HZO lying in the sample surface plane under the external bias. To provide the X-ray scattering from these particular planes, the incidence angle of the X-ray beam was set to 15° in the sample surface direction. The details of the utilized geometry are presented in Supporting Information, Section S3.

**Figure 7:**
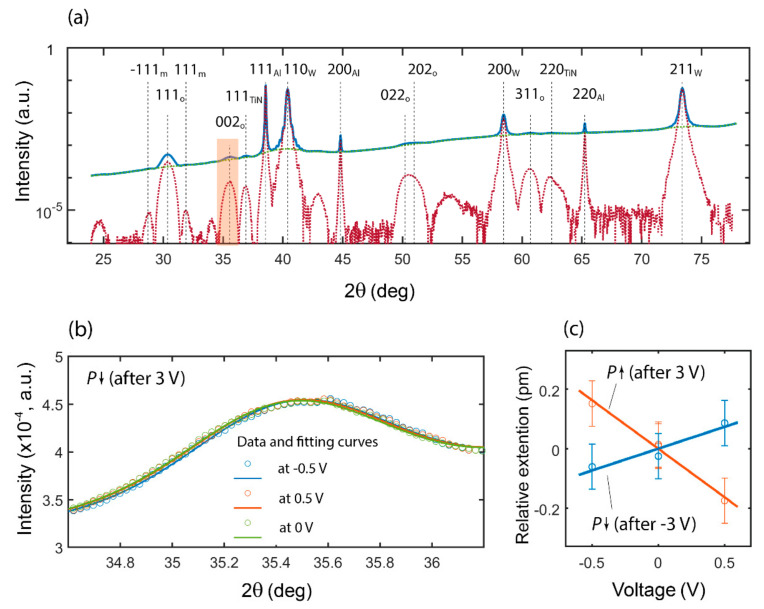
Measurement of the piezoelectric coefficient of the native HZO by in situ synchrotron X-ray microdiffractometry (micro-XRD). (**a**) Full spectrum (002_o_ peak is highlighted by orange; blue solid line is original spectrum, green solid line is a background, red dotted line is spectrum with subtracted background). (**b**) raw spectra and their fitting curves in the 2θ range 34–36° at different applied voltages; (**c**) relative extension of *d*-spacing for two monodomain polarization states.

In Figure 7a, the full XRD spectrum with identified peaks is shown, whereas in Figure 7,b the XRD spectra and fitting curves in the 2θ range 34–36° specific for the 002_o_ reflections of the HZO are presented for the downward polarization state at three different bias voltages. At first sight, the spectra obtained under varying external bias only negligibly differed from each other. However, there was a slight monotonic shift in the 002_o_ position and, therefore, in the *d*-spacing, with a change in the bias in both polarization states.

In Figure 7c, the relative extensions of the *d*-spacing that were calculated as the change in the *d*-spacing related to the *d*-spacing at 0 V dependent on the applied bias for both the up- and the downward polarizations are presented. The first finding is that a piezoelectric effect with a negative piezoelectric coefficient took place. Indeed, one can see that the extension rose when the applied bias was aligned opposite to the polarization vector direction. Next, the effective piezoelectric coefficients, calculated as the slopes of the presented dependences, differed in two polarization directions: −0.33 ± 0.03 pm/V for the upward polarization and 0.15 ± 0.04 pm/V for the downward polarization. These values are extremely small compared to those obtained from similar experiments for the PZT-based capacitors (30–340 pm/V) [33]. The small values of the piezoelectric coefficients were related to the miniscule shift in the XRD spectra under varying bias (Figure 7b) compared to the easily observable difference in the case of the PZT [33]. It is noteworthy that the magnitude of the piezoelectric coefficient previously reported for ferroelectric hafnium oxide was an order of magnitude larger than that obtained for the HZO in our experiment.

The difference in the value of the effective piezoelectric coefficient obtained for the two monodomain polarization states may have been due to different reasons. First, it may have been due to the more stable upward polarization, i.e., to upward nanodomains that persisted in the downward polarization state. The aforementioned effect of the mechanical coupling between the adjacent domains could have caused the decrease in the measured effective piezoelectric coefficient. Another probable origin is the different in-plane mechanical stress within the HZO film in h=the two polarization states. Moreover, the doping might have changed the in-plane stress and, thus, the doped HZO might have the piezoelectric coefficient of another magnitude and sign compared to the native film.

#### 3.3.2. The Piezoelectric Coefficient Measured by Means of BE PFM

Since the in situ XRD experiment revealed a negative sign of the piezoelectric coefficient of the native HZO, the opposite switching of the PFM phase in the native and doped regions of the HZO could have been associated with a different sign of the piezoelectric coefficient, i.e., the native film had a negative piezoelectric coefficient, while the Ga-doped HZO with a dopant concentration of 0.1 at.% had a positive piezoelectric coefficient.

**Figure 8:**
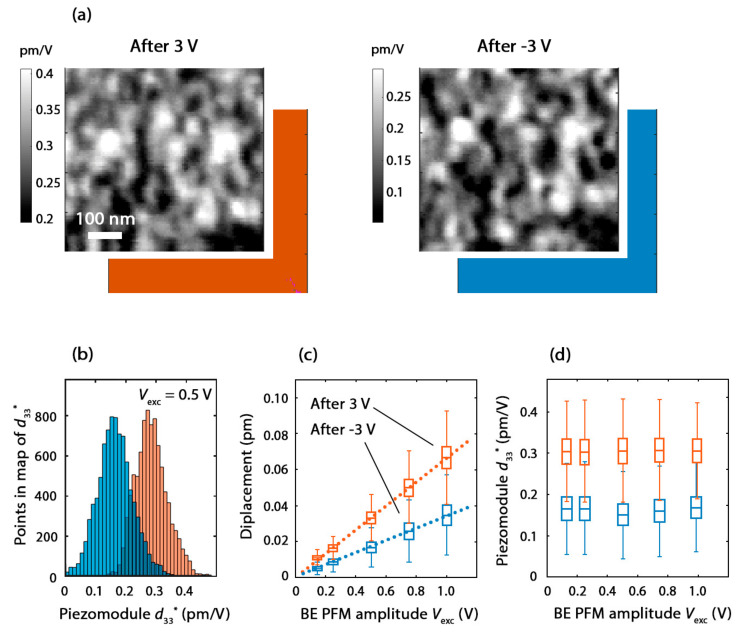
Distribution of the effective piezoelectric coefficient *d*_33_* for two polarization states. (**a**) Maps of spatial distribution of the piezoelectric coefficient and PFM phase at excitation voltage with amplitude *V*_exc_ = 0.5 V. (**b**) Distributions of piezoelectric coefficient over regions in (**a**) at BE PFM amplitude 0.5 V. (**c**) Dependences of vertical displacement on BE PFM amplitude with box plot (statistical-type parameters of distribution of displacement over corresponding maps); (**d**) dependences of piezoelectric coefficient on BE PFM amplitude with box plot. In (**b**–**d**) orange color corresponds to upward polarization, blue color corresponds to the downward polarization.

To acquire the distribution of the magnitude of the piezoelectric coefficient over the native HZO, we mapped the effective piezomodule *d*_33_* for both the upward and the downward polarization states by means of BE PFM. The PFM phase maps provide the uniform distribution of the vertical component of the polarization vector (Figure 8a). For the quantitative measurement of the local piezoelectric coefficient magnitude, the PFM amplitude measured in our PFM setup in arbitrary units needed to be calibrated in length units, i.e., in meters. The most reliable calibration method is based on fitting the spectral thermal noise of the cantilever deflection near the free resonance frequency [34]. The distribution of the piezomodule in the PFM maps clearly exhibited the different magnitudes of the effective piezoelectric coefficients of the two polarization states: −0.31 ± 0.05 pm/V and −0.17 ± 0.04 pm/V. These values were very close to those obtained in the XRD experiment, except that here, ± 0.05 pm/V and ± 0.04 pm/V were not the error, but the standard deviation of the distribution of the piezoelectric coefficient over the area 550 × 550 nm^2^ in size.

It is now clear why the amplitude on the SS–PFM curves differed at different voltage polarities (Figure 5a). It was associated with a different effective piezoelectric coefficient for the two polarization states.

The piezoelectric coefficient was so small that it seemed to be close to the electrostriction coefficient. Indeed, the electrostrictive displacement Δ*d*_z_ of the capacitor plates in the HZO capacitor was due to Hook’s law: $\Delta d_{z}=F_{z}/k_{zz}$, where *F*_z_ is the attraction force between the plates at a given bias voltage, *k_zz_* is the force constant of the HZO layer, equal to the *zz*-component of the force-constant tensor, *z* is the vertical axis that is normal for the capacitor plates. Since $k_{zz}=\frac{ES}{d}$ (where *E* is Young’s modulus, *S* is the area of the HZO capacitor, and *d* is the thickness of HZO layer) and $F_{z}=\frac{W}{d}=\frac{CV^{2}}{2d}$ (where *W* is the energy of the capacitor, $C=\frac{\varepsilon_{0}\varepsilon S}{d}$ is its capacity, *V* is the bias voltage, and ε is the dielectric permittivity), the electrostrictive displacement is $\Delta d_{z}=\frac{\varepsilon_{0}\varepsilon V^{2}}{2dE}$. At *E* = 150 GPa [35], ε = 40 (this work), *d* = 10 nm, and $\Delta d_{z}$ is 0.12 and 0.03 pm for bias voltages of 1 and 0.5 V, respectively. In other words, the electrostrictive displacement seems to be numerically comparable to the measured piezoelectric coefficient.

Since the electrostrictive displacement had a quadratic dependence on the bias voltage, it was generally expected that would not contribute to the PFM results given the zero *dc* component of the electric field in the HZO layer. In real capacitors, a small *dc* component of the electric field *V_DC_* could be present, although the external electric field is not applied—due to potential differences between the top- and bottom-electrode materials. Thus, there could have been some electrostrictive contribution to the PFM data.

To examine the contribution of electrostriction to the measured piezoelectric coefficient, we verified the dependence of the measured surface displacement on the amplitude of the excitation voltage. For both polarizations, it was found to be accurately linear (Figure 8c). The piezomodule *d*_33_* was found to be constant at different amplitudes of the excitation voltage (Figure 8d). These results prove that electrostriction did not affect our PFM results and the PFM amplitude in our experiment accurately corresponded to the piezoelectric coefficient of the HZO.

Notably, the displacements measured by the PFM were very small (0.005…0.07 pm, Figure 8c), and they seemed to be even smaller than the electrostriction displacements. However, there was no contradiction, because in the BE PFM technique, the amplitude *V*_exc_ of the drive voltage waveform corresponds to the voltage per frequency harmonic equal to $V_{\mathrm{exc}}/\sqrt{2N}$, where *N* = 1024 is the number of harmonics in the spectrum of the excitation signal (Supporting Information, Section S4). Therefore, the displacements in Figure 8c correspond to dc voltage, which was ~45 times smaller than the BE PFM amplitude.

By means of both synchrotron XRD and PFM, it was shown that the effective longitudinal piezoelectric coefficient of the native HZO was negative and that its value differed for the two polarization states: −0.30 and −0.15 pm/V for up- and downward polarizations, respectively. The lightly (0.1 at.%) Ga-doped HZO was in a polydomain state even after voltage pulses of −3.2/3.2 V, and the doped regions contained domains with opposite PFM-phase switching to that of the native film. The opposite switching was associated either with a positive piezoelectric coefficient or with ferroelastic switching. Both of these phenomena are determined by mechanical strain, which depends on the density of the oxygen vacancies formed due to the doping of transition metal oxides. Furthermore, the different piezoelectric coefficient for the two polarization states may also have been associated with different mechanical strains due to the non-equivalent interfaces with the electrodes. From the standpoint of applications, the changes in the sign of the piezoelectric coefficient and the ferroelastic switching were similar to each other; however, from the point of view of fundamentals, additional studies are required to differentiate between them.

## 4. Conclusions

In summary, we demonstrated a new ferroelectric hafnium oxide thin film, specifically, Ga-doped HZO, and compared its ferroelectric and piezoelectric properties with those of undoped HZO. The lightly doped HZO exhibited an improved switchable polarization, whereas doping with larger dopant concentrations resulted in a natural deterioration in ferroelectric properties.

To study the relationship between the measured polarization, the local ferroelectric properties, and the local piezoelectric properties, we employed the approach of local doping implemented via the local implantation of Ga ions into an amorphous 10nanometer-thick HZO film by means of a focused ion beam. Subsequently, annealing was used for the local crystallization of the HZO:Ga film. Comparing the ferroelectric properties of the Ga-doped HZO thin films with the results of the piezoresponse force microscopy, we found that the largest switchable polarization was accompanied by partial switching in the domain structure, while the smaller switchable polarization of the native HZO corresponded to full domain switching. Therefore, ferroelectric film, which is desirable for implementation of FeRAM, may not be the best choice for FeFET and FTJ applications. Moreover, at the largest switchable polarization, an apparently anomalous switching of the domain structure was observed, which was intertwined with the piezoelectric properties of the film, namely, with the sign of the piezoelectric coefficient.

By comparing the results of the in situ piezoresponse force microscopy with the results of the in situ synchrotron X-ray microdiffractometry, we revealed that the native HZO had a negative longitudinal effective piezoelectric coefficient, and its value differed for two polarization states: ~0.30 and ~0.15 pm/V for the up- and downward polarizations, respectively. The highly doped HZO had an even smaller piezoelectric coefficient, which was supported by simulation. An apparently anomalous (opposite) switching was observed in the lightly doped HZO. The opposite switching was associated either with a positive piezoelectric coefficient or with ferroelastic switching, which were both induced by the mechanical strain modulated by the oxygen vacancies formed due to the doping of the transition metal oxides. The results of our work indicate that diverse ferroelectric and piezoelectric properties can be achieved in HZO by means of the engineering of the oxygen vacancy density and, thus, by the engineering of mechanical strain.

## Data Availability

Not applicable.

## Supplementary Materials

The following supporting information can be downloaded at: https://www.mdpi.com/article/10.3390/nano12091483/s1. Section S1: Simulation of gallium implantation; Section S2: Voltage train during the measurement of SS-PFM loop; Section S3: Geometry utilized in the synchrotron microXRD experiment; Section S4: Quantitative calibration of band excitation PFM amplitude to vertical displacement and piezomodule in absolute units [36].
