# Supplementary material for: Nanoscale Doping and Its Impact on the Ferroelectric and Piezoelectric Properties of Hf0.5Zr0.5O2
Source: Nanomaterials (Basel). 2022 Apr 27;12(9):1483. doi: 10.3390/nano12091483 (PMC9103790; doi:10.3390/nano12091483)
Supplement: Supplementary file 1 [file nanomaterials-12-01483-s001.zip › nanomaterials-1682716-supplementary.pdf]

Supplementary Materials

Nanoscale doping and its impact on ferroelectric and piezoelectric properties of $Hf_{0.5}Zr_{0.5}O_2$

Anastasia Chouprik, Roman Kirtaev, Evgeny Korostylev, Vitalii Mikheev,*
Maxim Spiridonov, Dmitrii Negrov

Section S1. Simulation of Gallium Implantation

Prior to implantation of gallium ions this process was simulated in SRIM/TRIM software (Stopping and Range of Ions in Matter / Transport of Ions in Matter) using 3-layer model (Table S1) of TiN/$Hf_{0.5}Zr_{0.5}O_2$/TiN stack. Implantation was performed in a thin-film capacitor geometry, i.e. ions were implanted through top electrode, specifically, 10 nm-thick titanium nitride film. The primary result of TRIM calculation is depth distribution of stopped Ga ions that shows how much atoms left in HfO_2. To maximize this fraction, 30 keV beam energy was used – simulations showed (Figure S1) than 60% of total Ga^+ are stopping in hafnium oxide; e.g. for 20 keV the part is 43%, for 10 keV – 8% and at 5keV all ions left in top TiN layer.

Table S1. Layer stack for TRIM simulations

Layer	Thickness	Density	Elements (atom# / mass in amu)	Stoichiometry
TiN (top)	10 nm	5.64 g/cm^3	Ti (22/48)	0.500
			N (7/14)	0.500
$Hf_{0.5}Zr_{0.5}O_2$	10 nm	9.68 g/cm^3	Hf (72/178)	0.167
			Zr (40/91)	0.167
			O (8/16)	0.666
TiN (bottom)	20 nm	5.64 g/cm^3	Ti (22/48)	0.500
			N (7/14)	0.500

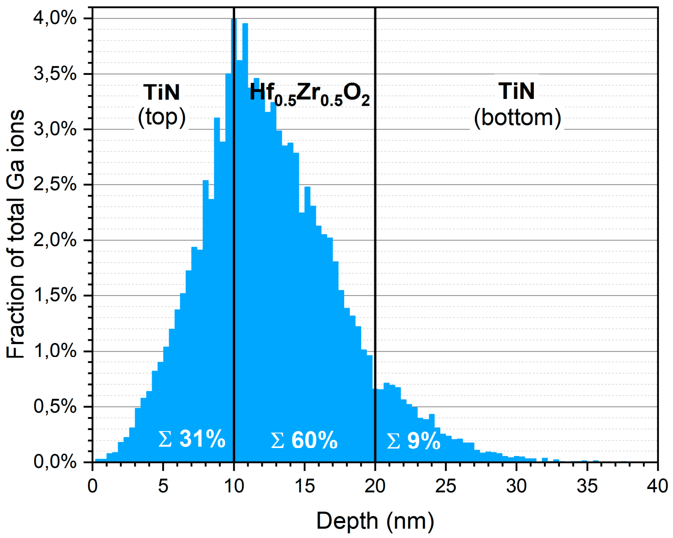

Figure S1. Simulated distribution of Ga atoms across thin film capacitor.

Using calculations above doses could be converted to ions/cm^2:

$$D_{Hf_{0.5}Zr_{0.5}O_2} = 0.60 * D_{total}$$

$$D\left[\frac{\text{nC}}{\text{um}^2}\right] = \frac{10^{-9} * 6.25 * 10^{18}}{(10^{-4})^2} * D\left[\frac{\text{ions}}{\text{cm}^2}\right] = 6.25 * 10^{17} * D\left[\frac{\text{ions}}{\text{cm}^2}\right]$$

or to atomic Ga concentrations:

$$C = \frac{D\left[\frac{\text{ions}}{\text{cm}^2}\right]}{N}$$

where N is number of atoms in 1 cm^2 of 10 nm thick hafnium-zirconium oxide layer:

$$N = \frac{V * \rho * N_A}{M} = 3 * \frac{(1^2 * 10 * 10^{-7}) * 9.68 * \ 6 * 10^{23}}{0.5 * 178.5 + 0.5 * 91.2 + 2 * 16} = 1.04 * 10^{17} \text{ atoms}$$

Built-in into system vector scanning module is utilized to write desired patterns. Pattern is loaded to control software as a bitmap image; herewith the size of structures is set up by choosing a magnification of FIB scanning system. Module allows to expose any dose from 10^{-4} nC/um^2 with step 10^{-4} nC/um^2. Typical exposed doses are listed in Table S2.

Table S2. Calculated ion doses for set of Ga concentrations

**Total dose, nC/um^2**	**Doses into $Hf_{0.5}Zr_{0.5}O_2$**		
	**nC/um^2**	**Ions/cm^2**	**At. %**
0.0002	1.2E-04	7.5E+13	0.07
0.0007	4.2E-04	2.6E+14	0.32
0.0011	6.6E-04	4.1E+14	0.50
0.0015	9.0E-04	5.6E+14	0.68
0.0019	1.14E-03	7.1E+14	0.86
0.0023	1.38E-03	8.6E+14	1.04

0.0029	1.69E-03	1.1E+15	1.32
0.0035	1.90E-03	1.2E+15	1.48

Section S2. Voltage Train during the Measurement of SS-PFM Loops

The single-switching PFM (SS-PFM) curves were obtained in the off-field mode [36] locally, i.e., every SS-PFM curve corresponded to the switching of the single grain under the AFM tip. The piezoresponse was excited and measured after passing of the switching voltage pulse (Figure S2) at zero *dc* voltage.

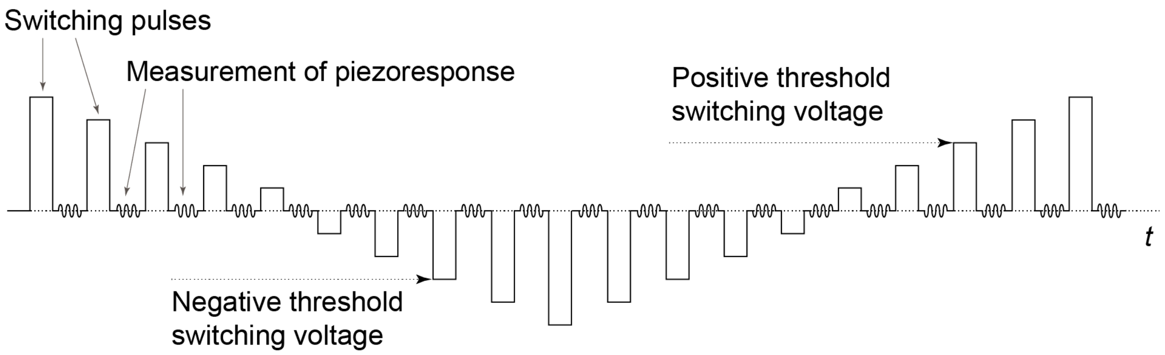

Figure S2. Schematic voltage train during PFS measurement and the indication of the moments of the anomalous switching.

Section S3. Geometry Utilized in the Synchrotron microXRD Experiment

The simplest way to estimate the effective piezoelectric coefficient along the normal surface direction by *in situ* synchrotron X-ray micro-diffractometry is to analyze the evolution of *d*-spacing between [002] planes of $Hf_{0.5}Zr_{0.5}O_2$ lying in the sample surface plane under the external bias. To provide the X-ray scattering from these particular planes, the incidence angle of the X-ray beam was set to be 15 deg to the sample surface direction (Figure S3).

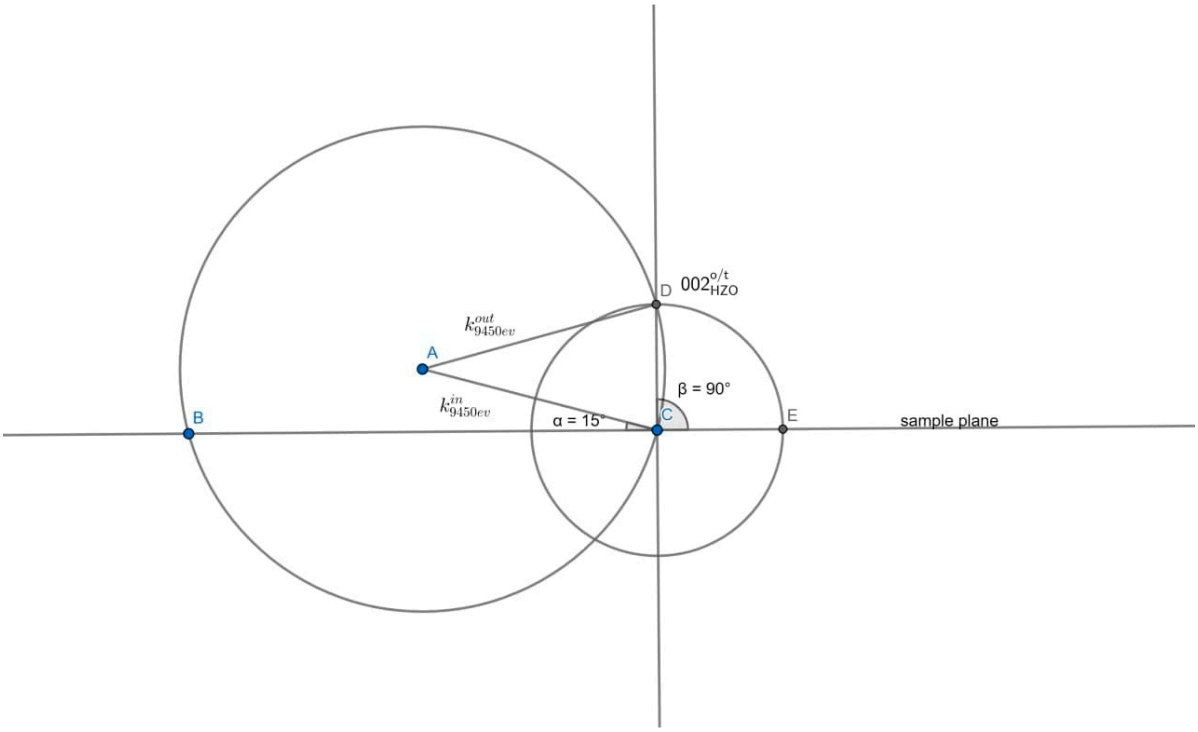

Figure S3. Geometry in the experiment on the measurement of the piezoelectric coefficient by *in situ* synchrotron X-ray micro-diffractometry.

Section S4. Quantitative Calibration of Band Excitation PFM Amplitude to Vertical Displacement and Piezomodule in Absolute Units

For the measurement of the piezomodule, the PFM amplitude *A* measured in AFM Ntegra in arbitrary units should be calibrated to units of length, i.e. to meters. The most reliable calibration method is based on fitting of the spectral thermal noise of a cantilever near a free resonance frequency.

A cantilever that is not deliberately excited but in equilibrium with a thermal bath at temperature *T* exhibits random fluctuations resulting in the measurable noise of the cantilever displacement signal. Furthermore, all electrical and optical components that are part of the AFM detection system produce noise, superimposed on the displacement signal. Therefore, the power spectral density D_{tot} $\left[\left(\mathrm{m}/\sqrt{\mathrm{Hz}}\right)^2\right]$ of the total displacement signal noise can be described as

$$D_{\mathrm{tot}} = D_{\mathrm{th}} + D_{\mathrm{ds}}, \qquad \text{(S1)}$$

where D_{th} and D_{ds} represent the thermal and the detection system contribution. The power spectral density of thermal noise in the resonant system is defined by formula [32]:

$$D_{\mathrm{th}}(f) = \frac{2k_B T / (\pi k_0 f_0 Q_0)}{\left(1-(f/f_0)^2\right)^2 + \left(f/(f_0 Q_0)\right)^2} \qquad \text{(S2)}$$

where k_B is Boltzmann constant, f_0 is the first eigenfrequency of cantilever beam, k_0 is the force constant of a cantilever, Q_0 is the quality factor of the cantilever oscillation.

The power spectral density of the detection system noise of AFM NTegra used in current experiment is defined as [S3]:

$$D_{\mathrm{ds}}(f) = \frac{1}{(d+hf)^2}, \qquad \text{(S3)}$$

where h and d are parameters of slope and shift of frequency dependence of detection system noise.

For calibration of the PFM amplitude, fitting of the spectral thermal noise was performed using formula (S1). Since the commercial software of AFM Ntegra does not provide sufficiently large number of averaging of the absolute amplitude spectra to achieve the obvious thermal peak of cantilever, we used custom software, which has unlimited possibility of averaging. Besides, for acquisition of both thermal noise peak and piezoresponse spectra we used the band excitation PFM technique [34] home-implemented in AFM Ntegra [24]. Given definite width of frequency bin, fitting of noise spectra $A(f)$ by (S1) using the basin-hopping optimization algorithm provides optimal values of f_0, k_0, Q_0, h, d . Further, we subtract the detection system contribution from the total spectral density, compare extracted pure resonance-enhanced thermal noise [a.u.] to theoretical value [m] and achieve calibration coefficient K [m/a.u.], which means a cantilever sensitivity.

In Figure S4 the result of fitting of the experimental noise absolute amplitude spectrum is shown. For a cantilever used for the PFM study in this work, we obtained following values for free parameters of fitting model: f_0 = 111 kHz, Q_0 = 314, k_0 = 5.7 N/m. Noise level of our AFM setup (corresponding to green line in Figure S4) in amplitude units was found $\sqrt{D_{\mathrm{ds}}} = 130\ \mathrm{fm}/\sqrt{\mathrm{Hz}}$.

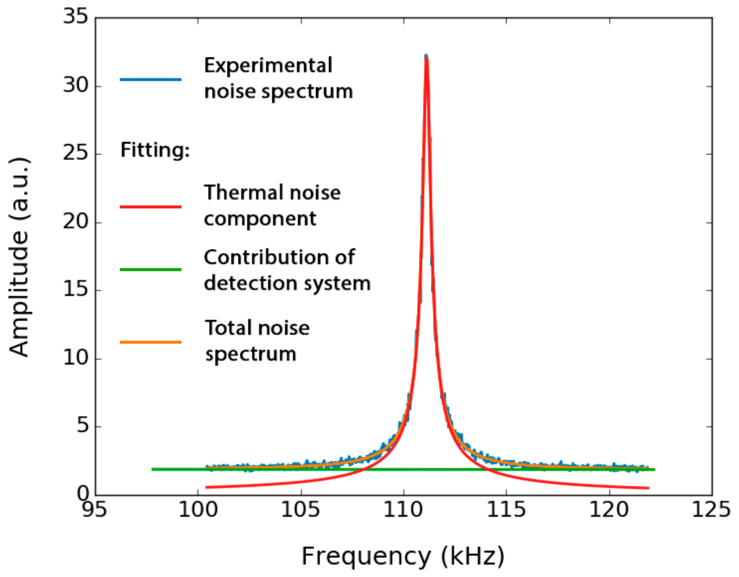

Figure S4. Experimental noise absolute amplitude spectrum and the result of its fitting by formulas (S1)-(S3).

For the piezoresponse obtained by the resonance-enhanced PFM, the static ("low-frequency") vertical displacement [m] is defined as

$$D = \frac{A_0 K}{Q_0}, \tag{S4}$$

where A_0 is resonance amplitude of piezoresponse in a.u.

For the band excitation resonance-enhanced PFM, the piezomodule [m/V] is defined as

$$d_{33}^{\mathrm{eff}} = \frac{D}{\Delta V} = \frac{A_0 K}{Q_0\ \Delta V}, \tag{S5}$$

where ΔV is the voltage per the one frequency harmonic (the voltage per the frequency bin).

The voltage per the frequency bin is equal to

$$\Delta V = \frac{V_{\mathrm{exc}}}{\sqrt{2N}}, \tag{S6}$$

where N is the number of frequency harmonics, V_{exc} is half of the peak-to-peak value of the excitation voltage in the time domain. The excitation voltage waveform depends on chosen phase spectrum; this issue is discussed in [24]. For $N = 1024$ chosen in our experiment, $\Delta V = V_{\mathrm{exc}}/46$.

In general form, for the band excitation resonance-enhanced PFM, the piezomodule is calculated in following way

$$d_{33}^{\mathrm{eff}} = \frac{\sqrt{2N} D}{V_{\mathrm{exc}}} = \frac{\sqrt{2N} A_0 K}{Q_0\ V_{\mathrm{exc}}}. \tag{S7}$$
